# Supplementary material for: A rapid and accurate approach for prediction of interactomes from co-elution data (PrInCE)
Source: BMC Bioinformatics. 2017 Oct 23;18:457. doi: 10.1186/s12859-017-1865-8 (PMC5654062; doi:10.1186/s12859-017-1865-8)
Supplement: Additional file 1: — Supplementary Figures and Tables. (DOCX 4945 kb) [file 12859_2017_1865_MOESM1_ESM.docx]

**Table S1.** Predicted interactions using CORUM, IntAct, or hu.MAP as gold standard reference. Numbers are the predicted interactions uniquely predicted by a single gold standard (“unique”), or commonly predicted between two gold standards, or common between all three gold standards. Numbers correspond to figures 3F and Supp. Fig. 4A-C.

| Dataset | CORUM  unique | IntAct  unique | hu.MAP  unique | CORUM  and IntAct | CORUM  and hu.MAP | IntAct  and hu.MAP | CORUM,  IntAct, and  hu.MAP |
| --- | --- | --- | --- | --- | --- | --- | --- |
| D1 *^a^* | 7504 | 11781 | 11 | 11985 | 5690 | 5555 | 5527 |
| D2 *^b^* | 2002 | 30213 | 0 | 5231 | 0 | 0 | 0 |
| D3 *^c^* | 1281 | 6054 | 0 | 4409 | 0 | 0 | 0 |
| D4 *^d^* | 5696 | 28528 | 0 | 10708 | 273 | 616 | 273 |

*a* Fig. 3F*, b* Supp. Fig. 4A*, c* Supp. Fig. 4B*, d* Supp. Fig. 4C

**Table S2.** Interaction score significantly correlates with other, independent evidence of protein interaction.

| Dataset | GO  CC | GO  BP | GO  MF | Proteome  Abundance | Subcellular  Localization | Structurally  Resolved |
| --- | --- | --- | --- | --- | --- | --- |
| D1 | 0.09  2.8e-35 | 0.22  6.3e-172 | 0.2  7.1e-142 | 0.24  4.8e-35 | 0.11  1.1e-36 | 0.12  9.6e-61 |
| D2 | 0.12  4.7e-21 | 0.23  3.7e-69 | 0.18  1.4e-43 | 0.26  6.8e-13 | 0.11  1.8e-13 | 0.21  1.1e-74 |
| D3 | 0.1  4.5e-12 | 0.12  2.9e-64 | 0.22  1.1e-48 | 0.26  3.8e-15 | 0.057  3.4e-4 | 0.13  1.2e-22 |
| D4 | 0.74  2.6e-17 | 0.26  2.8e-109 | 0.23  1.8e-82 | 0.24  6.9e-19 | 0.063  5.7e-10 | 0.12  2.5e-55 |

Spearman correlation coefficients (top) and p-values (bottom), corresponding to Figure 5AB, Supp. Figure 3AB.

**Table S3.** Interacting versus non-interacting enrichment values, PrInCE versus previously published interaction lists.

| Dataset | Software | Number  of inter-actions | GO  CC | GO  BP | GO  MF | Proteome  Abundance | Subcellular  Localization | Structurally  Resolved |
| --- | --- | --- | --- | --- | --- | --- | --- | --- |
| D1 | PrInCE | 11452 | 1.5  1e-10 | 8.3  <1e300 | 6.6  <1e-300 | 4.0  2.8e-79 | 2.5  1.4e-57 | 5.2  3.6e-280 |
| D1 | *a* | 11447 | 1.0  0.35 | 4.5  6.2e-206 | 4.8  <1e-300 | 3.6  2.9e-59 | 2.3  2.6e-81 | 4.4  2.4e-212 |
| D2 | PrInCE | 9409 | 1.5  1.2e-16 | 10.4  <1e-300 | 6.2  <1e-300 | 3.7  2.5e-78 | 2.3  3e-21 | 6.8  <1e-300 |
| D2 | *b* | 9411 | 1.8  1.7e-24 | 7.7  <1e-300 | 5.5  <1e-300 | 4  3e-64 | 1.5  1.7e-5 | 6.1  <1e-300 |
| D4 | PrInCE | 7205 | 1.4  7e-11 | 10.0  1e-300 | 6.6  <1e-300 | 5  3.4e-91 | 2.1  1.3e-10 | 5.9  2.3e-234 |
| D4 | *c* | 7209 | 1.8  1e-30 | 5.3  3e-235 | 4.7  1.1e-218 | 3.6  2e-32 | 1.2  0.06 | 5.0  1.9e-180 |

Fold enrichment (top) and hypergeometric p-values (bottom). PrInCE interactions lists were controlled to have the same number of interactions as previously published lists. *a* (Scott *et al.*, 2017), *b* (Scott et al., 2015), *c* (Kristensen *et al*. 2012)

**Figure S1.** Example distance measures for the same proteins in Figure 3. A. One minus the Pearson correlation coefficient, 1 *− Rcorr* . B. Corresponding p-value to 1 *− R*, *pcorr* . C. Euclidean distance, *E*. D. Peak location, *P*. E. Co-apex score, *CA*. See Methods for definitions.

**Figure S2.** Average number of interactions achieved at 50% precision using differently-sized subsets of each dataset.

**Figure S3.** For a large enough reference, PrInCE stably predicts the same core interactions. A. Number of predicted interactions using variable sized subsets of the CORUM complexes, evaluated at 50% precision, dataset D1. B. Same data as A, but expressed in terms of the number of gold standard pairs (PPIs) rather than number of gold standard complexes. C. Commonly predicted interactions are higher scoring. Predicted interactions were generated with 10 random subsets of 287 CORUM complexes (A, left side). D. Non-overlapping sets of CORUM PPIs used as references (see Methods). Only CORUM PPIs with both proteins quantified (dataset D1, single replicate) were counted. Numbers differ from B because only a single replicate was used. E. Interactions predicted using CORUM sets in D.

**Figure S4.** Number of predicted interactions and their overlap when using CORUM (purple), IntAct (blue), or hu.MAP (orange) for datasets D2 (A), D3 (B), and D4 (C). Interactions evaluated at 50% precision. hu.MAP is not shown in A and B because no interactions were predicted at 50% precision. D. Gold standard pairs from CORUM and IntAct have higher correlating chromatograms than pairs from hu.MAP.

**Figure S5.** Predicted interactions are enriched for biologically meaningful attributes, and the degree of enrichment reflects interaction score. A. Fraction of interacting proteins with at least one shared subcellular localization annotation as a function of interaction score. B. Fraction of interacting proteins with a structurally resolved domain-domain interaction as a function of interaction score. C. GO term Jaccard index distribution or non- interacting protein pairs and interacting pairs with a score *≥* 0.75 or between 0.5 and 0.75. Dataset D1.

**Figure S6.** A-C. GO term Jaccard index distribution in datasets D2 (A), D3 (B) and D4 (C) for non-interacting protein pairs and interacting pairs with a precision *≥* 0.75 or between 0.5 and 0.75. D-F. Interacting proteins in datasets D2 (D), D3 (E), and D4 (F) are enriched for shared GO-slim terms relative to non-interacting protein pairs at diverse GO term breadths. G-J. Fraction of interacting and non-interacting protein pairs coexpressed at or above a given tissue proteome abundance Pearson correlation coefficient (Kim et al., 2014) threshold between zero and one in datasets D1 (G), D2 (H), D3 (I), and D4 (J).

**Figure S7.** Distinct topological properties of high- and low-precision edges in datasets D1 (A-D), D2 (E-H), D3 (I-L), and D4 (M-P). Removing low-precision edges fragments the network into more (A, E, I, M) and smaller (B, F, J, N) connected components, results in a smaller largest connected component (C, G, K, O), and leaves fewer proteins connected (D, H, L, P). Grey regions show the average +/- one standard deviation.

**References**

Kim, M.-S., Pinto, S. M., Getnet, D., Nirujogi, R. S., Manda, S. S., Chaerkady, R., Madugundu, A. K., Kelkar, D. S., Isserlin, R., Jain, S., *et al.* (2014). A draft map of the human proteome. *Nature*, 509(7502), 575–581.

Kristensen, A. R., Gsponer, J., and Foster, L. J. (2012). A high-throughput approach for measuring temporal changes in the interactome. *Nature methods*, **9**(9), 907– 909.

Scott, N. E., Brown, L. M., Kristensen, A. R., and Foster, L. J. (2015). Development of a computational framework for the analysis of protein correlation profiling and spatial proteomics experiments. *Journal of proteomics*, 118, 112–129.

Scott, N. E., Rogers, L. D., Prudova, A., Brown, N. F., Fortelny, N., Overall, C. M., and Foster, L. J. (in press). Interactome disassembly during apoptosis occurs independent of caspase cleavage. *Molecular Systems Biology*.
